# NH_2_ Measurements during the Pyrolysis and Oxidation of Ammonia/Propane Mixtures in a Shock Tube

**DOI:** 10.1021/acs.jpca.6c04551

**Published:** 2026-10-06

**Authors:** Matthew Abulail, Cade W. Cash, Olivier Mathieu, Eric L. Petersen

**Affiliations:** J. Mike Walker’66 Department of Mechanical Engineering, 14736 Texas A&M University, College Station, Texas 77843, United States

## Abstract

Ammonia/propane pyrolysis and oxidation mixtures were studied using NH_2_ laser spectroscopy to obtain NH_2_ time histories in a shock tube. An NH_3_ diagnostic based on a quantum cascade laser was used to verify the initial ammonia concentrations, while a 597.375-nm laser diagnostic measured NH_2_ in the post-reflected shock region. A 75/25% (by volume) ammonia/propane pyrolysis mixture, diluted in 99% argon, was tested between 1682 and 2926 K at near-atmospheric pressure. Three elementary reactions—C_3_H_8_ ⇆ C_3_H_7_ + H, NH_3_ + H ⇆ NH_2_ + H_2_, and NH_2_ + H ⇆ NH + H_2_—were identified as influential for accurately modeling NH_2_. Additionally, mixtures composed of 75/25% ammonia/propane with O_2_ in 99% argon were tested at equivalence ratios of 0.5, 1.0, and 2.0 between 1318 and 2404 K. Four key reactions, namely C_3_H_8_ + M ⇆ C_2_H_5_ + CH_3_ + M; NH_3_ + H ⇆ NH_2_ + H_2_; NH_3_ + OH ⇆ NH_2_ + H_2_O; and O_2_ + H ⇆ O + OH, heavily influenced the production of NH_2_ at early time scales, with CH_4_ + M ⇆ CH_3_ + H + M and H_2_ + O ⇆ H + OH being responsible for the delayed destruction of NH_2_. The data presented in this study are available in the supplementary material for chemical kinetics modeling validation.

## Graphical abstract

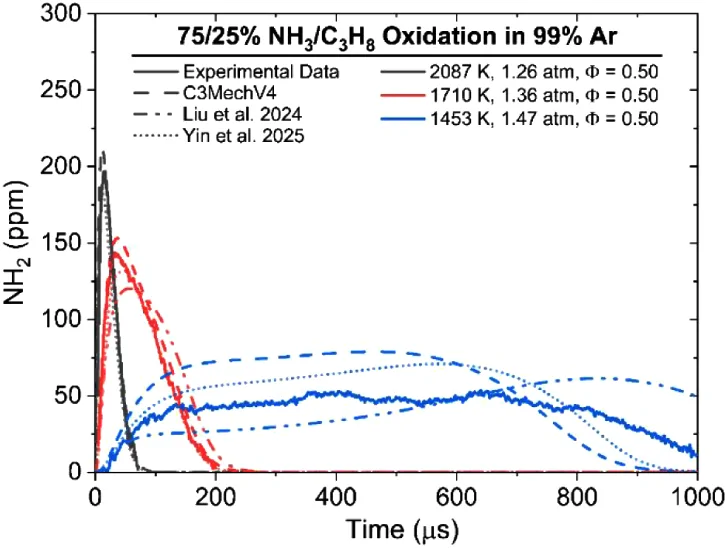

## Introduction

Because of interest in its carbon-free nature, the industrial-scale production of ammonia, and its ease of storage compared to hydrogen,^1^ a great deal of research has been devoted to ammonia combustion over the past decade. While more work is still necessary to fully comprehend the combustion chemistry of ammonia,^2–4^ it is well documented that ammonia has poor combustion properties, with long ignition delay times^5^ and low flame velocities^6^ compared to traditional fuels. As a result, blends of ammonia with more reactive fuels have been considered. Mixtures of H_2_ and NH_3_, for example, have been studied extensively as a carbon-free solution (the H_2_ being possibly produced by the thermal cracking of ammonia).^7–10^ Methane, the main component of natural gas, is also often considered in mixtures with ammonia as a low-carbon solution^8,11,12^ because of its high H/C ratio and because it can also be produced from biomass. Other fuel-ammonia mixtures have been considered as well, such as blends with methanol,^13–15^ DME,^16–18^ etc.

Another hydrocarbon of particular interest for mixing with ammonia is propane, because it is a large component of liquefied petroleum gas (LPG), a common fuel with a high H/C ratio. Propane is reactive and has a high energy density, facilitating ammonia utilization through combustion enhancement. Propane also has a boiling point and a liquefaction pressure similar to those of ammonia, facilitating transport, storage, and use in dual-fuel, direct-injection applications such as industrial burners and furnaces^19,20^ or large engines.^21^ As such, the combustion chemistry of ammonia/propane mixtures has received some attention recently.

For instance, the laminar flame speed of various NH_3_/C_3_H_8_ mixtures has been measured in several studies using various techniques.^22–24^ Speciation studies have also been performed in a jet-stirred reactor by Yin et al.^25,26^ and Liu et al.^27^ Additionally, Lin et al.^28^ measured the ignition delay time of NH_3_/C_3_H_8_ mixtures in shock tubes with varying propane concentrations. Ignition delay times were also measured in a rapid compression machine by Liang et al.^29^ for a wide range of high pressures, 20–100 bars. A new detailed chemical kinetic model was also proposed by Liu et al.^27^ and validated against previous literature data.^21–25^ A recent study by Pratali Maffei et al.^30^ includes ammonia chemical kinetic data with further propane-related improvements derived from the NUIG 1.1 model used by Yin et al.^25^ The practical aspect of NOx formation from NH_3_/propane co-combustion was studied in a swirl burner by Wu et al.^19^ Recent attempts at ammonia integration into propane-based spark-ignition engines by Żółtowski and Gis^31^ have shown moderate success, with an additional study by Haputhanthri et al.^32^ demonstrating further advances with ammonia-gasoline blends.

As can be seen, the experimental database for the combustion of ammonia/propane mixtures is growing but remains limited and, aside from the speciation data reported by Yin et al.,^25,26^ mostly comprises global kinetics data only. The goal of the present study was to complement this database by providing NH_2_ time-history profiles during the pyrolysis and oxidation of 25/75% C_3_H_8_/NH_3_ mixtures in a shock tube. First, the methodology and diagnostics used to study these mixtures are described, followed by a presentation of the results and their comparison with detailed kinetic models.

## Methodology

The Aerospace Shock Tube (AST) at Texas A&M University was used exclusively for this study. The AST is a high-purity, stainless-steel, pressure-driven shock tube. The driven (low-pressure) and driver (high-pressure) sections feature 16.2- and 7.62-cm inner diameters and 7.88- and 3.25-m lengths, respectively. The two sections are separated by a Lexan polycarbonate diaphragm, which is designed to deflect until the desired pressure is reached, upon which it is pierced by a custom die cutter, bursting the diaphragm and initiating the test. The incident shock is tracked by pressure transducers spaced across the last 1.56 m of the driven section, with the last pressure transducer located 1.6 cm from the end wall. The pressure increase from the incident shock at each transducer location is used to obtain the incident-shock velocity. The shock velocity, mixture composition, initial temperature, and pressure are used in the normal-shock relations to obtain the post-incident- and post-reflected-shock temperature and pressure, with uncertainties of ±0.8% and ±1%, respectively.

All mixtures were prepared in a stainless steel mixing tank using the partial pressure method. Various combinations of ammonia, propane, oxygen, and argon were used for the mixtures, with purities (by vol.) of 99.995%, 99.97%, 99.999%, and 99.999%, respectively. A targeted blend of 75/25% ammonia-to-propane was used in this study since the peak NH_2_ concentrations are reliant on the main production pathway from NH_3_, thus requiring a higher ammonia concentration than what might be expected when integrated into an application. Passivation of the mixing tank was done prior to every mixture by inserting ammonia for 10 min. The initial ammonia pressure was recorded after 10 min, and this recorded pressure was used for the remainder of the mixture calculations to represent the partial pressure of NH_3_ in the mixing tank. The gas manifold was vacuumed using a turbomolecular pump after each gas addition to ensure high-purity mixtures.

Passivation also occurs during experiments to minimize the effects of ammonia loss due to adsorption within the AST. The AST is evacuated using a turbomolecular pump prior to passivation, after which some of the mixture compositions are introduced into the shock tube. This study used 10 Torr for passivation of the pyrolysis mixture and 30 Torr for passivation of the propane-ammonia oxidation (stoichiometric and rich) mixtures. Since the concentration of ammonia in the lean equivalence ratio propane-ammonia oxidation case was very small, passivation used 60 Torr of mixture to ensure adequate saturation of the stainless steel. The mixture was left in the shock tube driven section to passivate for 10 min and was then evacuated to 0.08 Torr. The turbomolecular pump was not used during this step, because the strength of the vacuum produced could result in the desorption of ammonia. Finally, the desired initial test pressure of the mixture (P_1_) is introduced into the driven section, and the test is commenced.

Direct ammonia measurements were taken using the ammonia quantum cascade laser-based diagnostic stationed at 957.839 cm^–1^ developed by Alturaifi et al.^33^ to verify the ammonia concentration. The system uses spectroscopic data presented by the HITRAN database^34^ and Alturaifi et al.^35^ to obtain an absorption coefficient. The full set of calculations for obtaining the absorption coefficient is detailed in Alturaifi et al.,^33^ with an absorption coefficient uncertainty estimated to be ±12%. The Beer-Lambert relationship, shown in eq 1, is used to obtain the mole fraction (*X_NH_*_3_) based on the ratio of light transmission across the post-test region (*I*_t_/*I*_0_), absorption coefficient (*k_v_*), test region path length (*L*), and pressure (*P*). The modification included in eq 2 adjusts the light intensity in eq 1 to include detector biases $(\Delta_{I_{0}}$ / $\Delta_{I_{0}-I_{t}}$ ), and the initial offset voltage (*δ*_*initial*_).

1 $$\ln\left(\frac{I_{t}}{I_{0}}\right)=-k_{v}PX_{NH_{3}}L$$

2 $$\frac{I_{t}}{I_{0}}=1-\frac{{\left(I_{0}-I_{t}\right)}_{measured}-\delta_{initial}}{\left({\left(I_{0}\right)}_{measured}-\Delta_{I_{0}}\right)-\left(\delta_{initial}-\Delta_{I_{0}-I_{t}}\right)}$$

Every variable defined in eqs 1 and 2 is obtained from spectroscopic data available from HITRAN^34^ and Alturaifi et al.,^35^ except for the difference in voltage between photodetectors, *δ_initial_*. When measuring non-passivating molecules, this term is usually adjusted until it matches the initial mixture concentration or an initial concentration of zero. The initial offset voltage can also be measured prior to an experiment; however, the inclusion of passivation, and the extended time needed for it, can lead to slight deviations in laser power and result in some *δ_initial_* deviation. An empirical modification of the Beer-Lambert relationship shown in eq 3 utilizes the change in absorption between the initial state and the post-incident shock to provide an accurate measurement of initial ammonia concentrations in the test section.

Calculating initial ammonia concentrations using this method provides two key benefits. First, the absorption of ammonia at pre- and post-incident shock conditions (17.3% and 28%, respectively) is significantly higher than at post-reflected-shock conditions (4.2%).^33^ Using the higher-absorption regions increases the signal-to-noise ratio used to predict the ammonia concentration, thereby providing higher accuracy. The second benefit of this empirical formula is that the initial and post-incident shock signals are taken as a ratio irrespective of the unknown *δ_initial_* , hence eq 3 minimizes the effect of initial offset voltage, so the initial ammonia measurement is purely based on spectroscopic data of ammonia and the signal-to-noise ratio.

3 XNH3=ln((ItI0)average,2*(I0It)average,1)L(P1kv,1−P2kv,2)

The NH_2_ diagnostic consists of a tunable diode laser operating at 1194.75 nm. The laser (MOGLabs Littrow) is fiber-connected to a second-harmonic generation unit that outputs a 597.375 nm free-space beam. The 597.375 nm wavelength corresponds to a region of high NH_2_ absorbance reported by Dressler et al.^36^ and Green et al.^37^ The Beer-Lambert relationship shown in eqs 1 and 2 is still used; however, spectroscopic data for NH_2_ are currently unavailable; therefore, an absorption coefficient from Mertens et al.^38^ is used. The uncertainty associated with the NH_2_ laser diagnostic is heavily influenced by the uncertainty associated with the absorption coefficient. While Mertens et al.^38^ estimated an uncertainty of ±30%, recent studies by Clees et al.^39^ and Abulail et al.^40^ agree with Mertens et al. but with much lower uncertainties. Abulail et al.^40^ estimate an uncertainty of ± 15% for the absorption coefficient.

The other main source of uncertainty is the initial ammonia concentration measurement outlined in eq 3. The initial ammonia concentration is effective when the absorption between initial and post-incident shock conditions is significantly different, mainly as a result of high ammonia concentrations and the peak NH_3_ absorption occurring at ∼800 K at 957.839 cm^–1^. Post-reflection temperatures >2350 K have post-incident shock absorbance similar to the incident-condition absorbance. The low differential in signal between these regions increases the uncertainty in the initial ammonia measurement in which the initial ammonia concentration is set as the mixture ammonia concentration. The total, initial NH_3_ uncertainty of ± 15% herein accounts for the higher uncertainty associated with the initial ammonia concentration.

The Mertens et al. relation for absorption coefficient is valid for temperatures between about 1650 and 3200 K; however, this study investigated temperatures as low as 1300 K. Since the absorption coefficient is not validated for these lower temperatures, the uncertainty associated with the measurements at temperatures lower than 1650 K is significantly higher. A conservative estimate outlined by Abulail et al.^41^ specifies an absorption coefficient uncertainty of ±30%. Additional uncertainties of ±0.8% and ±1.0% are included for post-reflected-shock pressure and temperature, respectively. An overall uncertainty in concentration of ±20% is expected for NH_2_ experimental traces, with a ±30% uncertainty for NH_2_ results at T_5_ < 1650 K.

## Results

Ammonia-propane pyrolysis mixtures were made as a 75/25% blend in 99% argon and tested from 1682 to 2926 K under near-atmospheric conditions. A 75/25% ammonia-propane blend was chosen to ensure sufficient NH_2_ production during ammonia oxidation because, at low fuel equivalence ratios, the average initial ammonia concentration is ∼25% of that in the pyrolysis model.

Figure 1 depicts low-, intermediate-, and high-temperature condition cases compared with propane mechanisms from Liu et al.,^27^ Yin et al.,^25^ and Maffei et al.^30^ (labeled C3MechV4). These models were chosen to integrate propane and ammonia chemistry. Yin et al. and Maffei et al. overpredict NH_2_ concentrations and tend to be overly reactive, but the overall trend is well captured. A comparison of the NH_2_ peak concentrations is shown in Figure 2. Variations in the experimental peak NH_2_ are correlated with differences in postreflected shock pressures and variations in initial ammonia concentrations. All models are simulated under the exact experimental conditions, but the applied exponential fit does not highlight the variation well. The differences for Maffei et al., Liu et al., and Yin et al. relative to the experimental data at 2926 K are 25.2%, 44.3%, and 10.7%, respectively; at 1682 K, the differences are 36.1%, 51.3%, and 16.4%, respectively.

**Figure 1:**
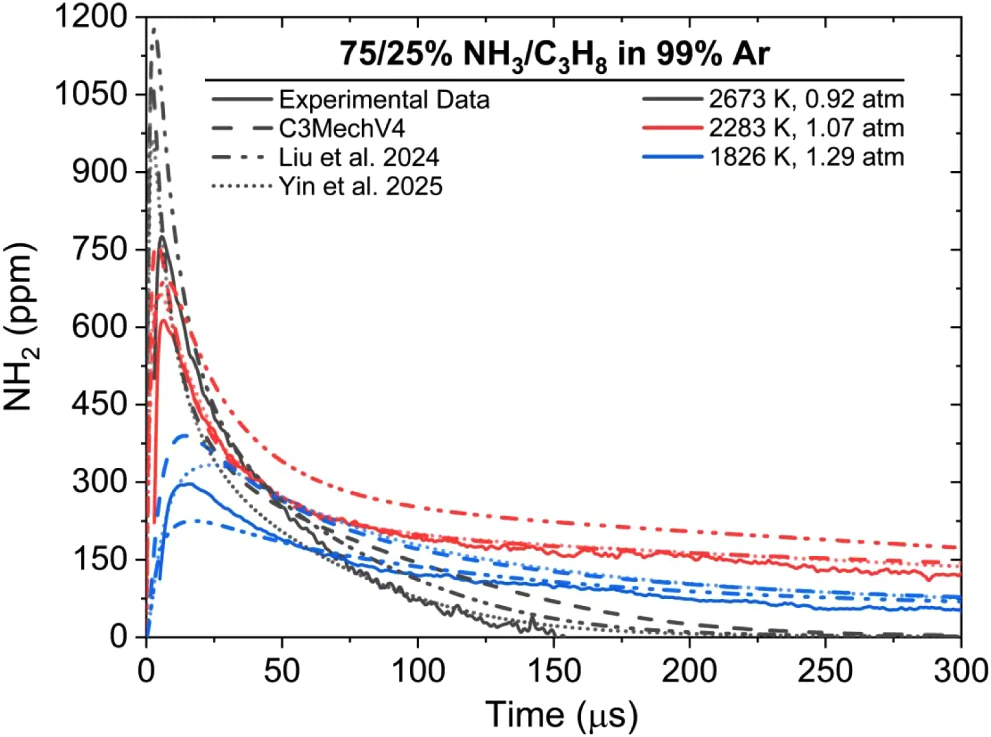
Three representative NH_2_ traces of ammonia-propane pyrolysis compared with Maffei et al.,^30^ Liu et al.,^27^ and Yin et al.^25^ Ten additional pyrolysis NH_2_ traces, not shown, are included in the supplementary material.

**Figure 2:**
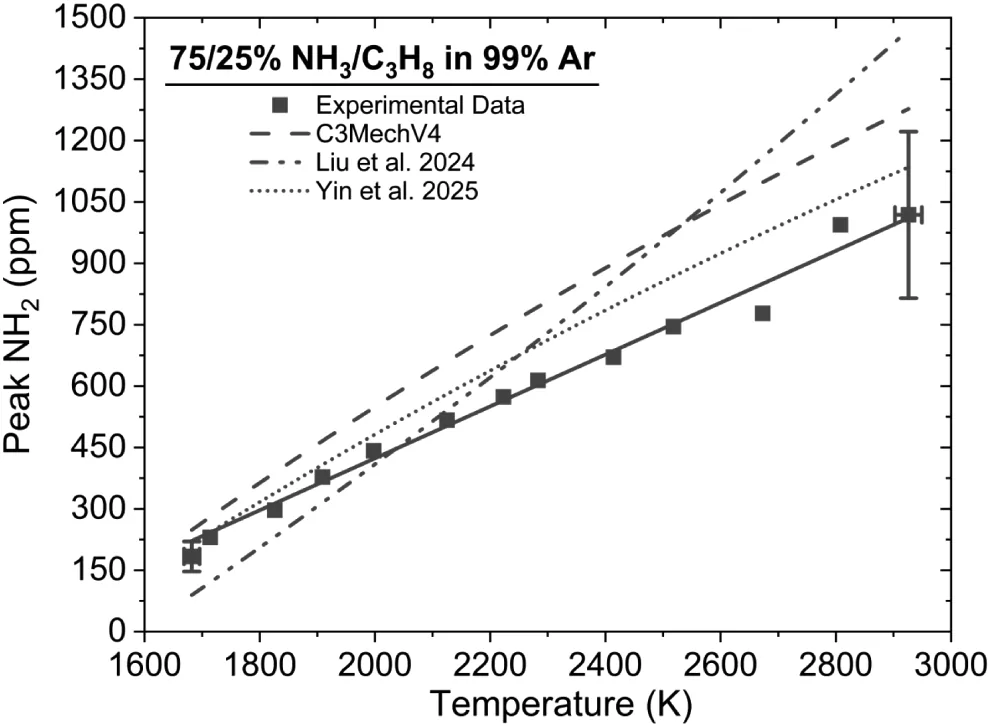
Peak NH_2_ comparison for ammonia-propane pyrolysis among experimental results, Maffei et al.,^30^ Liu et al.,^27^ and Yin et al.^25^.

Figure 3 presents the sensitivity analysis of the 2283 K, 1.07 atm pyrolysis simulation from Yin et al.^25^ The Yin et al. simulation is used for sensitivity analysis because of its higher accuracy in predicting peak ppm concentrations (Figure 2) and in the overall prediction of the NH_2_ trends (Figure 1). The NH_2_ initial production of NH depends on C_3_H_8_ ⇆ C_3_H_7_ + H (R1), which is the main producer of H atoms on early timescales. The H atoms produced then interact with ammonia through NH_3_ + H ⇆ NH_2_ + H_2_ (R2) to produce NH_2_, which immediately begins reacting again with H atoms through NH_2_ + H ⇆ NH + H_2_ (R3). All other reactions featuring the production and destruction of H atoms are minimal but together create an NH_2_ equilibrium in which production and destruction are balanced.

**Figure 3:**
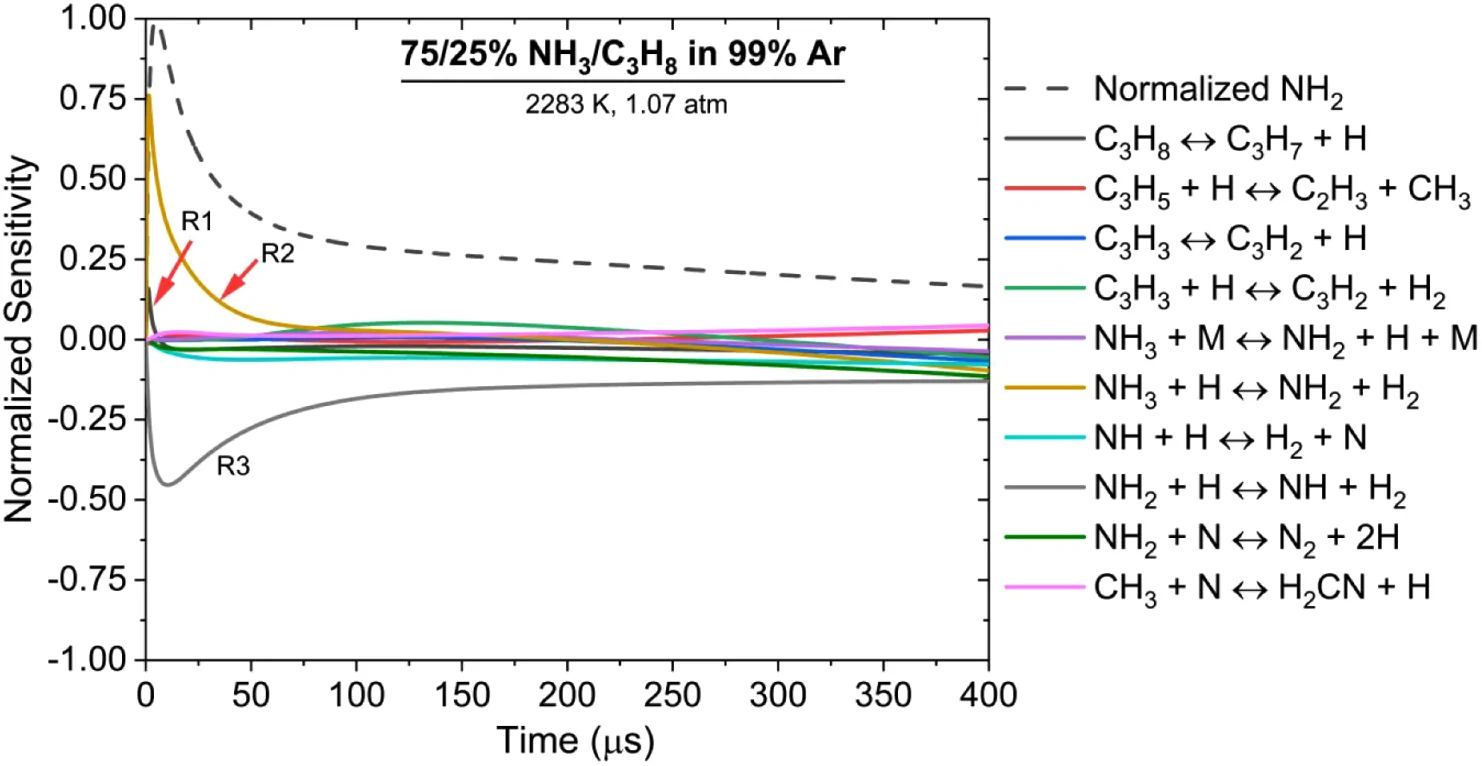
Sensitivity analysis of ammonia-propane pyrolysis at 2283 K and 1.07 atm, simulated with Yin et al.^25^.

Experiments on ammonia-propane with O_2_ at equivalence ratios of 0.5, 1.0, and 2.0 in 99% argon were also conducted. Low-, intermediate-, and high-temperature experimental traces are compared with Yin et al.^25^ and Maffei et al.^30^ in Figure 4 for equivalence ratios of 0.5, 1.0, and 2.0. C3MechV4 from Maffei et al. significantly overpredicts the NH_2_ concentrations, while Yin et al. accurately predict NH_2_ at the stoichiometric and rich equivalence ratios; they overpredict NH_2_ at the lean equivalence ratio. The high accuracy of Yin et al. is emphasized in the peak NH_2_ concentration measurements shown in Figure 5, where the peak ppm aligns directly with the experiments, except for lean experiments below 1600 K, where the peak ppm is overpredicted. The variations in the experimental peak ppm are subject to differences in initial ammonia concentrations and pressures.

**Figure 4:**
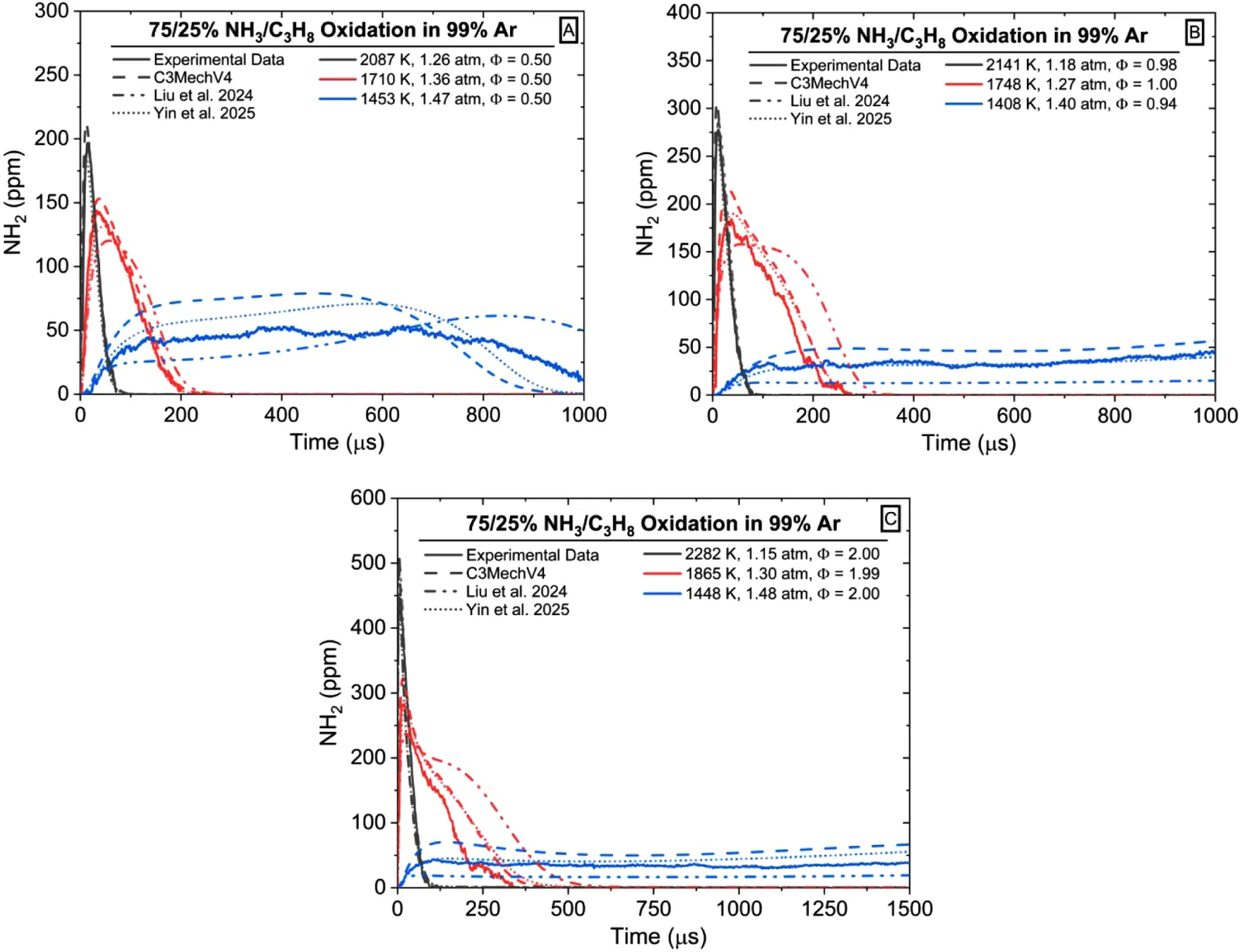
Comparison of low-, intermediate-, and high-temperature NH_2_ experimental traces across ammonia-propane oxidation at equivalence ratios of 0.5 (A), 1.0 (B), and 2.0 (C). Experimental traces are compared with Maffei et al.,^30^ Liu et al.,^27^ and Yin et al.^25^ Nineteen additional NH_2_ traces, not shown, across all oxidation mixtures are included in the supplementary material.

**Figure 5:**
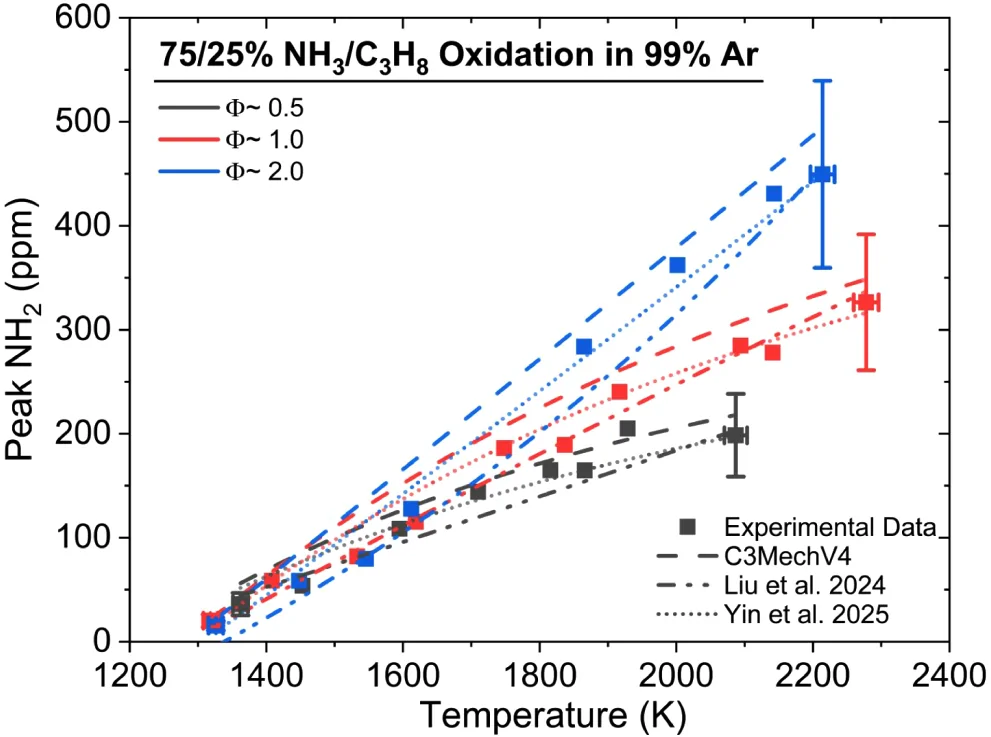
Peak NH_2_ comparison among experimental results, Maffei et al.,^30^ Liu et al.,^27^ and Yin et al.,^25^ at equivalence ratios of 0.5, 1.0, and 2.0.

The difference in performance between models is a result of limited validation for ammonia-propane mixtures. As mentioned by Maffei et al., C3MechV4 does include ammonia, but no validation for ammonia interactions with hydrocarbons is included.^30^ The accuracy shown by Yin et al. is a result of speciation data such as flow reactor measurements and laminar flame speeds, yet the deviation at lean equivalence ratios is a result of limited temporal speciation data and, perhaps, the older base mechanism of NUIG 1.1.^42^ The most recent version of NUIG at the time of Yin et al. was NUIG 1.3, which is heavily focused on propane oxidation and features improved model performance.^43^ The primary reason for Yin et al. to use NUIG 1.1 was the inclusion of ammonia chemistry. However, similar to C3MechV4, the ammonia-propane chemistry is not validated; thus, the ammonia-propane interactions rely on the ammonia chemistry as modified by Yin et al.

A unique trend captured by all mechanisms is the observation that there are three regimes of drastically different NH_2_ production and destruction, identifiable by the pseudo-plateau of NH_2_ concentrations after the peak, most evident in the 1865–K experiment at an equivalence ratio of 2.0 (Figure 4). Figure 6 shows the sensitivity analysis using Yin et al.^25^ for ammonia-propane oxidation at an equivalence ratio of 1.99 at 1865 K. The initial production of NH_2_ depends on 4 main reactions: R2, C_3_H_8_ + M ⇆ C_2_H_5_ + CH_3_ + M; (R4), NH_3_ + OH ⇆ NH_2_ + H_2_O (R5); and O_2_ + H ⇆ O + OH (R6). Key reactions in the initial NH_2_ production region depend on propane chemistry. Propane reacts at lower temperatures than ammonia;^33,43,44^ thus, the initial reactions from propane increase H and OH production (through R4 and R6), thereby stimulating the decomposition of ammonia at temperatures lower than 1650 K.^3,45^

**Figure 6:**
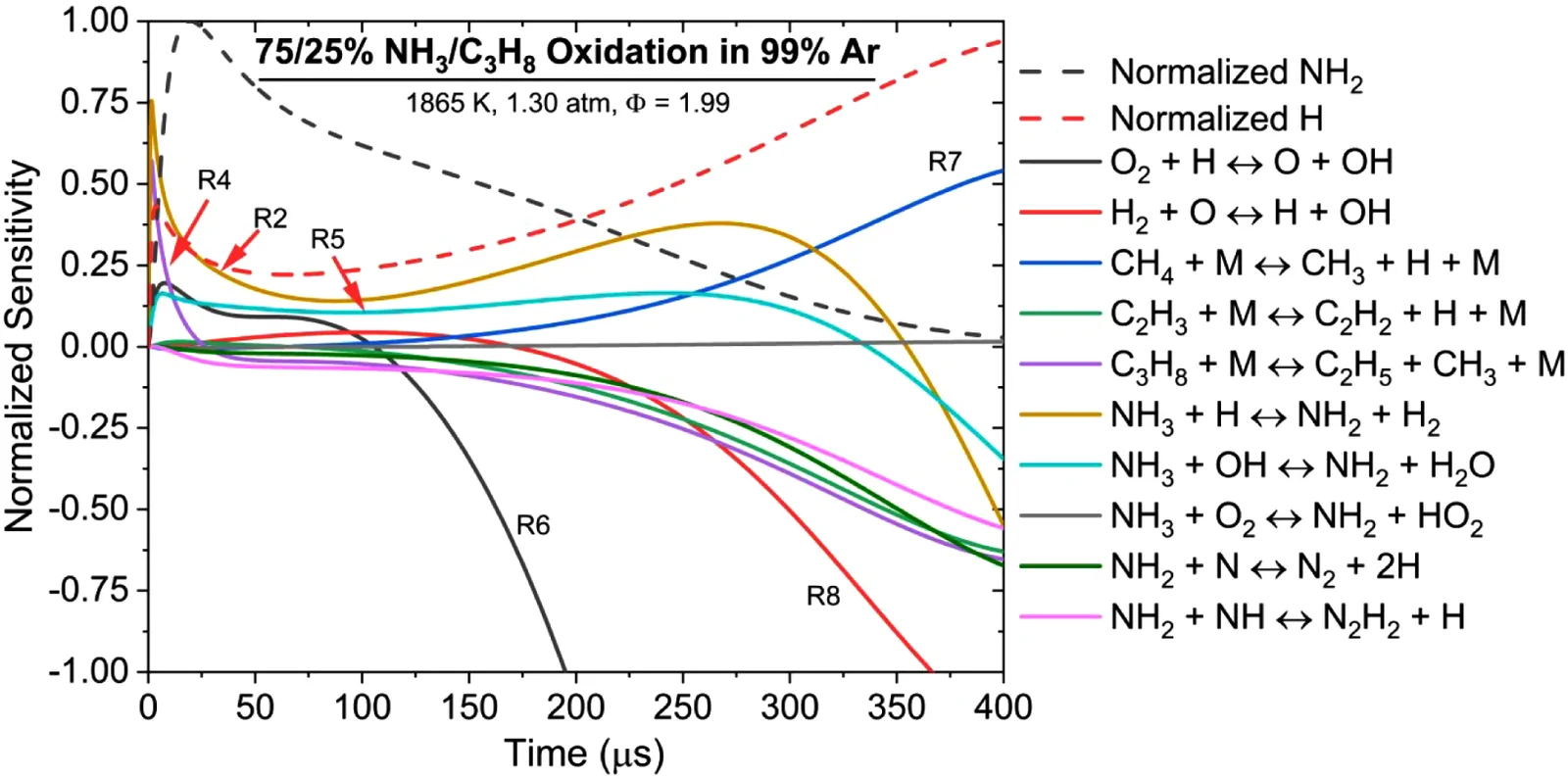
Sensitivity analysis of ammonia-propane oxidation at 1865 K and 1.3 atm, at an equivalence ratio of 1.99, simulated with Yin et al.^25^.

A highly atypical sensitivity associated with R2 indicates that the reaction’s sensitivity increases significantly after 100 μs, which occurs at the same time as the aforementioned pseudo-plateau of NH_2_. Figure 7 depicts the rate of production for Figure 6 and details the balance between R2 and R5, with the slow decay in production from R3 at the 100-μs timestamp. Hydrogen atom production is increased at that timestamp by propane decomposition into methane and subsequent methane decomposition into hydrogen through CH_4_ + M ⇆ CH_3_ + H + M (R7). The increase in H concentration leads to sustained NH_2_ production by R2 and R5. Reaction 2 produces H_2_ and thereby increases the impact of H_2_ + O ⇆ H + OH (R8) and, consequently, R5, yet other reactions, such as R6, compete with R5 and lead to the slow decay of NH_2_ rate of production via R5. Simultaneously, the concentration of ammonia is decreasing significantly, and thus the overall decomposition of NH_2_ accelerates toward zero concentration.

**Figure 7:**
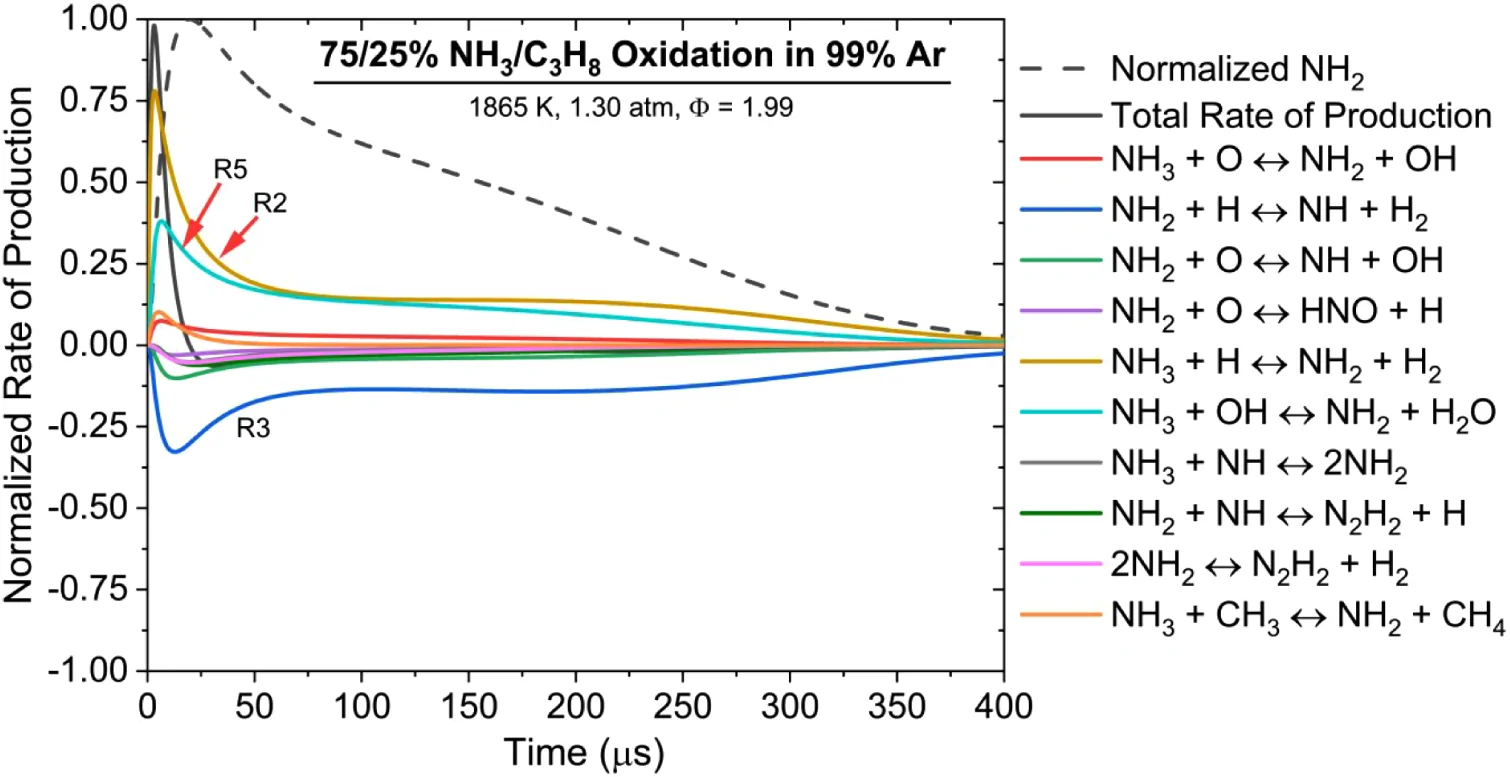
Rate-of-production analysis of ammonia-propane oxidation at 1865 K and 1.3 atm, at an equivalence ratio of 1.99, simulated with Yin et al.^25^.

Figure 8 presents the time-to-peak NH_2_ concentration compared with the models. A key trend shown across the pyrolysis experimental data is that the time to peak plateaus at temperatures above 2400 K. This behavior can be caused by the laser schlieren spike actively affecting the first 5 μs of the NH_2_ measurement (due to shock-wave passage); however, the peak concentration seen in Figure 2 shows excellent agreement across the experimental data, and this trend might be independent of the schlieren spike. The second key trend shown in Figure 8 is the two-stage time to peak observed in the oxidation data. Below 1750 K, the time to peak increases exponentially, so that each model is split into two lines to accurately display the relationship. Furthermore, the time to peak exceeded the maximum test time experienced in the shock tube at temperatures lower than 1700 K. The time to peak at the lower temperatures featured a distinct two-maxima trend in which the oxidation of propane initially creates NH_2_, which then begins to decay. The initial reaction of propane at lower temperatures increases the temperature in the shock tube, which promotes ammonia oxidation, thus resulting in a secondary overall production of NH_2_. This trend is evident in the low-temperature experimental and model traces in Figure 4. Maffei et al., Liu et al., and Yin et al. predict the transition in NH_2_ production behavior, yet the deviation from the peak ammonia concentrations and the inaccuracy across the models in Figures 1–2, 4–5, and 8 highlight that no current model accurately simulates NH_2_ time histories in ammonia-propane mixtures over the range of conditions in this study.

**Figure 8:**
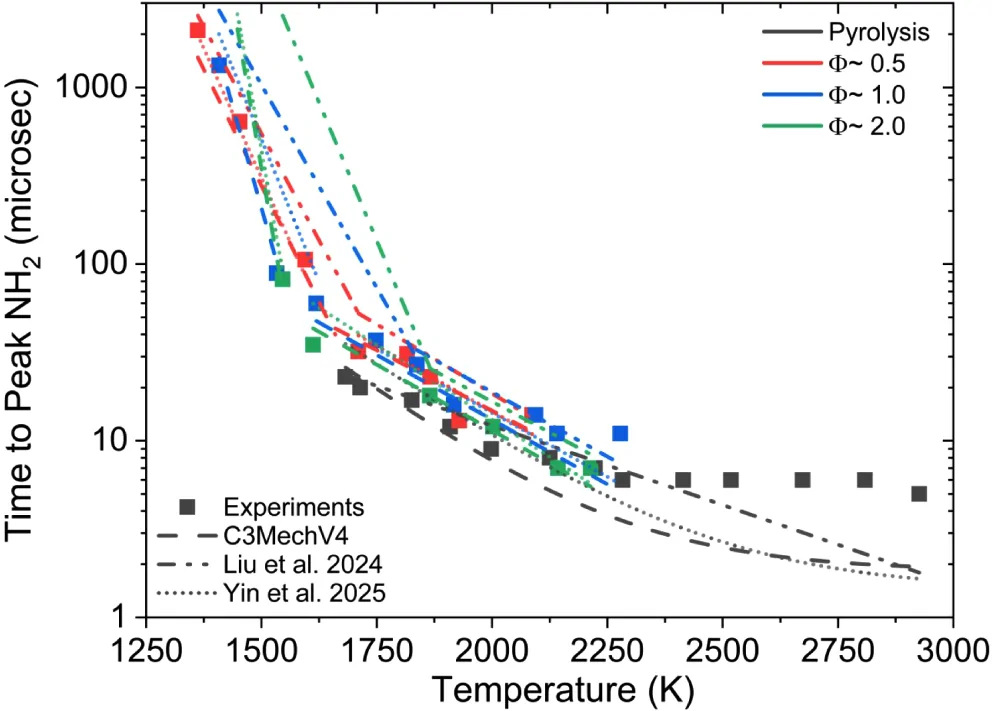
Time-to-peak NH_2_ from experimental data compared with Maffei et al.,^30^ Liu et al.,^27^ and Yin et al.^25^.

## Conclusion

NH_2_ concentrations were obtained for ammonia-propane pyrolysis and oxidation with O_2_ in a 99% argon dilution. Experimental traces for NH_2_ were recorded at 1682 to 2926 K for pyrolysis conditions and 1318 to 2404 K for equivalence ratios of 0.5, 1.0, and 2.0. The resulting data were compared with prominent models from the literature. Key reactions, C_3_H_8_ ⇆ C_3_H_7_ + H, NH_3_ + H ⇆ NH_2_ + H_2_, and NH_2_ + H ⇆ NH + H_2_ were primarily responsible for modeling NH_2_ in ammonia-propane pyrolysis, with the balance of NH_2_ production and destruction pathways resulting in a sustained concentration of NH_2_. Ammonia-propane oxidation mainly depends on NH_3_ + H ⇆ NH_2_ + H_2_, C_3_H_8_ + M ⇆ C_2_H_5_ + CH_3_ + M, NH_3_ + OH ⇆ NH_2_ + H_2_O, and O_2_ + H ⇆ O + OH for the initial production and destruction of NH_2_. Later timescales depend on hydrogen production from CH_4_ + M ⇆ CH_3_ + H + M, which increases NH_2_ through NH_3_ + H ⇆ NH_2_ + H_2_, thus delaying NH_2_ destruction.

## Supplementary Material



## Notes

S1: 39 additional NH_2_ experimental traces with initial conditions and ammonia concentrations (XLSX)
